# Combining survey data, GIS and qualitative interviews in the analysis of health service access for persons with disabilities

**DOI:** 10.1186/s12914-018-0166-2

**Published:** 2018-06-26

**Authors:** Arne H. Eide, Karin Dyrstad, Alister Munthali, Gert Van Rooy, Stine H. Braathen, Thomas Halvorsen, Frans Persendt, Peter Mvula, Jan Ketil Rød

**Affiliations:** 1 SINTEF, Department of Health, P.B.124, N-0314 Oslo, Norway; 2 Department of Sociology and Political Science, Norwegian University of Science and Technology, NO-7491 Trondheim, Norway; 3 Centre for Social Research, University of Malawi, P.O. Box 280, Zomba, Malawi; 4 Multidisciplinary Research Centre, University of Namibia, P. B. 13301, Windhoek, Namibia; 5 SINTEF, Department of Health, P.B. 4760, Torgarden, N-7465 Trondheim, Norway; 6 Department of Geography, History and Environmental Studies, University of Namibia, P.B. 13301, Windhoek, Namibia; 7 Department of Geography, Norwegian University of Science and Technology, NO-7491 Trondheim, Norway

**Keywords:** Health services, Vulnerable groups, Access, Low-income countries, Combining methods, Survey, Qualitative study, Geographical data

## Abstract

**Background:**

Equitable access to health services is a key ingredient in reaching health for persons with disabilities and other vulnerable groups. So far, research on access to health services in low- and middle-income countries has largely relied on self-reported survey data. Realizing that there may be substantial discrepancies between perceived and actual access, other methods are needed for more precise knowledge to guide health policy and planning. The objective of this article is to describe and discuss an innovative methodological triangulation where statistical and spatial analysis of perceived distance and objective measures of access is combined with qualitative evidence.

**Methods:**

The data for the study was drawn from a large household and individual questionnaire based survey carried out in Namibia and Malawi. The survey data was combined with spatial data of respondents and health facilities, key informant interviews and focus group discussions. To analyse access and barriers to access, a model is developed that takes into account both measured and perceived access. The geo-referenced survey data is used to establish four outcome categories of perceived and measured access as either good or poor. Combined with analyses of the terrain and the actual distance from where the respondents live to the health facility they go to, the data allows for categorising areas and respondents according to the four outcome categories. The four groups are subsequently analysed with respect to variation in individual characteristics and vulnerability factors. The qualitative component includes participatory map drawing and is used to gain further insight into the mechanisms behind the different combinations of perceived and actual access.

**Results:**

Preliminary results show that there are substantial discrepancies between perceived and actual access to health services and the qualitative study provides insight into mechanisms behind such divergences.

**Conclusion:**

The novel combination of survey data, geographical data and qualitative data will generate a model on access to health services in poor contexts that will feed into efforts to improve access for the most vulnerable people in underserved areas.

## Background

The human right to health [1] for all is enshrined in a number of international policy documents, including the Alma Ata Declaration [2], the 2030 Agenda for Sustainable Development [3], and the Convention on the Rights of Persons with Disabilities [4]. Equitable access to health services is a key ingredient in reaching health for persons with disabilities and other vulnerable groups. Nevertheless, existing research on access to health services for vulnerable groups in low- and middle-income countries is rather scattered, largely self-reported survey data, and to some extent inconclusive.

The purpose of this article is to describe and discuss an innovative methodological triangulation where statistical and spatial analysis of perceived distance and objective measures of access is combined with qualitative evidence from carefully selected areas. We aim to show that this approach provides a potentially stronger fundament for assessing access to health services in low-income contexts and yields new insight into lack of access, including the basis for individual perception of access. In our study, we focus explicitly on vulnerable groups, particularly people with disabilities. However, the approach can easily be adapted to other user groups.

There is an increasing recognition that access to health services and vulnerability vary geographically and individually. Where you live, who you are and with whom you live determine your access to health services. Although the interplay between physical environment and individual characteristics influences people’s access to health care, it is rarely taken explicitly into account in the theoretical framework or empirical design. Whether a physical factor, such as poor roads or long distance to nearest health facility, actually represents a barrier to access, depends on income, family situation and health. Thus, what merely constitutes an inconvenience for one person could represent an insurmountable barrier for someone else [5]. Arguably, the moderating factor here is vulnerability; local barriers are accentuated by individual characteristics that either alleviate or exacerbate access problems [6–9]. Therefore, we argue that to understand barriers to access, one should identify *circumstances* that lead to particularly poor access, i.e., situations where the combination of individual and contextual factors impedes access, explicitly taking the interplay between individual characteristics and people’s local environment into account. A handful of case studies by the World Health Organization (WHO) illustrate the usefulness of a spatial approach to access [10, 11]. In comparison, our approach also incorporates the subjective element of *perceived* access.

The studies by Trani et al. in Sierra Leone and Afghanistan [12, 13] have shown that there is a substantial gap between the perceived need for and received health services, and that persons with disabilities are disadvantaged regarding access when compared to non-disabled individuals. However, much of the literature has concentrated on identifying barriers, such as Van Rooy et al. [9] in Namibia, Vergunst et al. in rural South Africa [14], Munthali et al. [15] in Malawi, Eide et al. [16] and Visagie et al. [17] in four sub-Saharan countries. These studies and others confirm that there exists a range of environmental barriers to accessing health care and that a substantial number of persons with disabilities in low-income countries who need health services do not access it. This is a serious challenge in an equity perspective. To understand barriers to access, one should identify circumstances that lead to particularly poor access, i.e. situations where the combination of individual and contextual factors impedes access, explicitly taking the interplay between individual characteristics and people’s local environment into account.

While existing research is relatively congruent, few studies in low-income contexts have applied more objective measures such as distance, time of travel, topography and other environmental characteristics [18–22].

### Accessibility to health services

Accessibility to health services is a fuzzy term with a range of different definitions [23]. Access to health services is affected by contextual-, cultural-, community-, health service-, and individual level- characteristics as well as an interaction of these [24]. Access can further be split into components like availability, affordability, acceptability, adequacy, and accessibilty [25]. Access can, and mostly has been, measured as perceived access, through self-reported and subjective experience of access [26]. Access can also be measured as actual access, through directly observable dimensions like time and distance. Within health geography, spatial or geographical accessibility can be understood as the spatial separation of the population and the supply of health care facilities [11]. The other, non-spatial components to health access (affordability, appropriateness and accommodation) are commonly excluded from GIS (Geographical Information Systems) -based analysis.

Comparing geographical accessibility with perceived access and barriers to access can shed light on the strengths and limitations of different measurement as well as providing new insights into variations in the individual experiences of access to health care [11]. Ultimately, this knowledge can be used for improved planning and policy development.

### Access to healthcare for vulnerable people in resource poor settings in Africa

While the World Disability Report (WDR) [27] states that a large number of individuals with disabilities do not have access to health and other services (page xxi) and are denied equal access to health care (page 9), evidence is limited and mostly based on subjective measures. Data on access to health care in the 2002–2003 World Health Survey, which is a main source for WDR, is entirely based on individual perception of access. This is the case also for other studies, such as SINTEF’s studies on living conditions among persons with disabilities in low-income contexts (see e.g. [28]), and the Equitable study in four sub-Saharan countries [16, 17]. According to Eide et al. [16], lack of transport, poor availability of services, inadequate drugs or equipment, and cost of the visit are the four most pronounced barriers in both Namibia and Malawi.

The research project *EquitAble* **(**Enabling universal and equitable access to healthcare for vulnerable people in resource poor settings in Africa) was carried out over the period 2009–2012 by an international research consortium studying access to health services for vulnerable groups, including persons with disabilities. A comprehensive household survey was carried out in four African countries. In two of them, Namibia and Malawi, the data collection also included the GPS (Global Positioning System) coordinates of the sampled households. The sample comprises 1624 individuals in Namibia and 1526 individuals in Malawi, of which around 50 % were screened and identified as persons with disabilities using the Washington Group screening questions [29]. The selection of study sites in each country aimed at including populations with different characteristics and at the same time highlighting country specific characteristics, in this case a highly dispersed population (Namibia) and a population living in chronic poverty and with a high disease burden (Malawi). In Namibia, selected sites were Khomas (Central region), Hardap (south), Omusati (north), Kunene (north-west), and Caprivi (far north east). In Malawi, the four sites were Blantyre and Phalombe district (Southern region), Ntchisi (Central region) and Rumphi (Northern region). Clusters within each site were defined by the country teams based on the predefined characteristics as well as practical considerations.

*EquitAble* developed a framework for analysing human rights and inclusion in health policies [30], which led to the identification of a range of vulnerability factors that may influence on individual capabilities and ability to access services, including disability, ethnic minorities, female-headed households, limited resources (poverty), increased relative risk for morbidity, mother child mortality, children with special needs, aged, youth, displaced populations, living away from services, and suffering from chronic illness. By means of GPS coordinates and available geographical data, this provides an opportunity to analyse the relationship between perceived access and measured access and how this is influenced by vulnerability factors.

Building on *EquitAble,*^1^ an independent follow-up project, *GeoHealthAccess*^2^ – The geography of vulnerability and health service access in southern Africa – has set out to expand the empirical approach for access to healthcare by combining the geo-referenced survey data on perceived access, with geographical features like distance and travel time to health facilities. Notably, the project will combine quantitative analysis with qualitative data collection and analysis, where the selection of study sites for the qualitative data collection is guided by the quantitative analysis. The qualitative case studies serve to explore potential divergence between subjective and more objective measures of access.

### Health geography

Traditionally, there has been a divide between health geography and medical geography where the former uses qualitative approaches (as well as elaborating on social theories) while the latter uses quantitative methods (including GIS) and, largely, being atheoretical [31]. An emerging trend in health geography is to apply GIS in health promotion, planning and evaluation of health systems [32, 33]. A growing literature is devoted to physical access and how it should be measured, and complex models of spatial accessibility have been developed [32, 34, 35]. The suitability of GIS in improving health care in Africa and other parts of the world was pointed out almost a decade ago [36, 37]. Yet, despite the obvious inherent advantages in using GIS to map and plan access at the micro level, the application of these methods in Africa has been limited [37], in particular relative to Europe [38]. A recent literature review from South Africa found that health geography is still a limited research field [39], and there is little reason to believe that the progress has been more substantial in other African contexts. Traditionally, only GIS specialists have been able to model physical access to health care services, but this is being democratized with the general availability of GIS tools and georeferenced health care data. WHO has supported and financed this development, and made modelling of physical accessibility more available by the tool AccessMod [40]. Over the past decades, geographical research has taken on new ways, incorporating a triangulation of methods and knowledge, using both cartographic and non-cartographic information, such as surveys, epidemiological and qualitative data [39]. This approach is in line with the way the public health field engages with geography and space as described by Gatrell and Elliott [41]:

Modern public health sees the environment as social and psychological, not merely as physical. In this sense, then, “environment” and “place” converge to provide a spatial context for health that transcends the individual’s own behaviour and health outcomes (p. 15).

### Geography and perception

Within healthcare geography, several studies recognize the importance of including non-spatial factors, like demographics and socioeconomic status which influence access [33]. So far, this has largely implied aggregation of individual or household level characteristics to units or clusters. Despite several studies illustrating the usefulness of a GIS approach to health care [42–45], GIS applications to the study of health care and health outcomes in Africa remain limited. In addition, a weakness in most of them is that they do not take people’s *perception of access* into account. One exception is Manguno [46], who combined physical distance to the nearest immunization centre, with mothers’ perceptions of distance as determinants of child immunization in Nigeria, where perception of distance turned out to be a more robust determinant of access than actual distance. This finding highlights the need to combine more objective measures of accessibility with people’s perceptions of access to health care.

Furthermore, a weakness in the existing literature on individuals’ perception of access to health services in low-income contexts is the lack of data and in particular representative data that can be used to analyse accessibility more broadly. The complexity of measuring access may have influenced researchers to utilize barriers as a proxy to access. However, as pointed out by Fortney et al. [26], perceived access may be equally valid as a measure of access than more objective measures based on characteristics of health care systems. Individuals’ own interpretation of access, whether based on own or others’ experiences, knowledge, prevailing attitudes or other social or individual factors may influence on individuals’ health seeking behaviour and for instance whether to access health care services or not.

## Methods

### A mixed methods design: combing survey data on perceived access, GIS-based geographical measures and qualitative interviews

Building on these insights, *GeoHealthAccess* will develop and test a model of access to health care services for persons with disabilities in resource poor settings. The model combines physical accessibility with perceptions of access, individual level characteristics and vulnerability factors. The main hypothesis is that *there is a relationship between local context and access to health care, which is strengthened or mediated by vulnerability factors at the individual level*. The project combines *EquitAble* survey data on perceived access to health care with geographical data on distance and travel time to health facilities. GPS coordinates of households were recorded during data collection in Namibia and Malawi. Additionally, we will carry out qualitative studies to explore divergence between the survey and geographical data. During the first stage of *GeoHealthAccess*, we supplemented the household coordinates with the coordinates of the health clinics that each household uses (obtained from Ministry of Health). Together, these data form the basis for developing GIS-based measures of access. In the second stage, we use a statistical model of perceived access that combines the survey data and geographical data to identify areas where there are significant clustering of households with divergence in perceived access and GIS measured access. In the third stage, we revisit a strategic sample of these clusters to collect additional data that can explain such divergences. Here we conduct qualitative interviews and apply participatory GIS techniques.

### Empirical approach

The *EquitAble* household survey included data on demographics, perceived barriers to access health services and GPS coordinates of households. The novelty of *GeoHealthAccess* is the combination of *EquitAble* data at both household and individual level with additional geographical data and new qualitative data. To analyse access and barriers to access, *GeoHealthAccess* developed a model that takes into account both measured and perceived accessibility (using data from *EquitAble*). Table 1 presents possible outcomes of convergence and divergence between measured accessibility and perceived access.

**Table 1:**
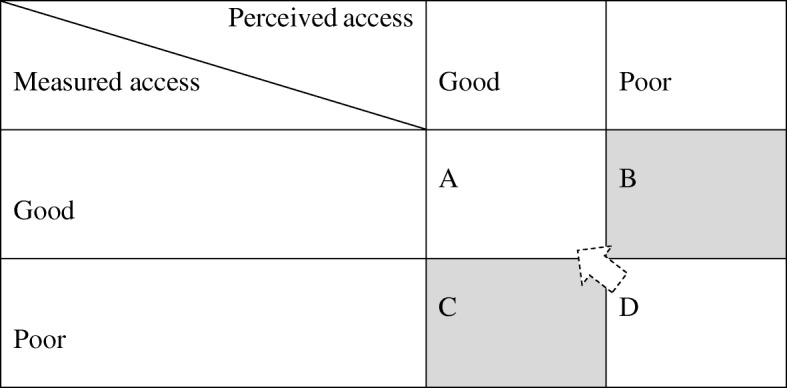
Possible outcomes of perceived and measured access

Outcome A and D in Table 1 represent full convergence of measured and perceived access, whereas C and D represent full divergence. The last two are theoretically interesting and represent an opportunity to explore the relationship between perceived and measured access. For instance, analysing differences in perceptions of access in outcomes C and B may reveal variation in individual characteristics and vulnerability factors that can drive the divergence in different directions.

To classify outcomes, we combined the geo-referenced *EquitAble* survey data with spatial features. The survey data comprised a series of questions about access and barriers to health services, socio-demographic variables as well as vulnerability factors that will be utilised to calculate perceived distance. The relationship between the need for and actual access to health services was measured in two different ways: i) a direct question on whether access was achieved the last time the respondents needed health services, and ii) a series of questions on awareness, need and access which can be used to calculate gaps in health services. These variables established whether perceived access was good or poor. Combined with an analysis of the terrain and the actual distance from where the respondents live to the health facility they go to, the data allows us to categorise areas and respondents according to the classification in Table 1. The four groups will then be analysed with respect to variation in individual characteristics and vulnerability factors. This will indicate underlying causes of discrepancies between perceived and measured access. While the table presents the four categories as dichotomous, in reality they are continuous phenomena that will add to the complexity of the analyses. By using various statistical techniques such as spatial regression analysis, structural equation modelling and multilevel analysis, we will be able to test preliminary models of access to health care.

**Fig. 1:**
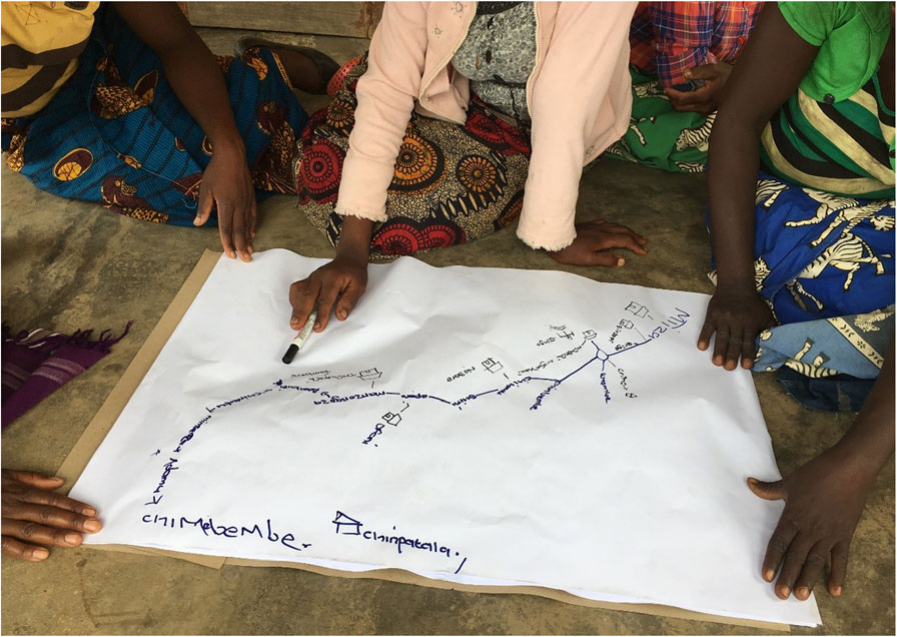
Participatory GIS/map drawing, Malawi 2017. Photo: Stine H Braathen, SINTEF

**Fig. 2:**
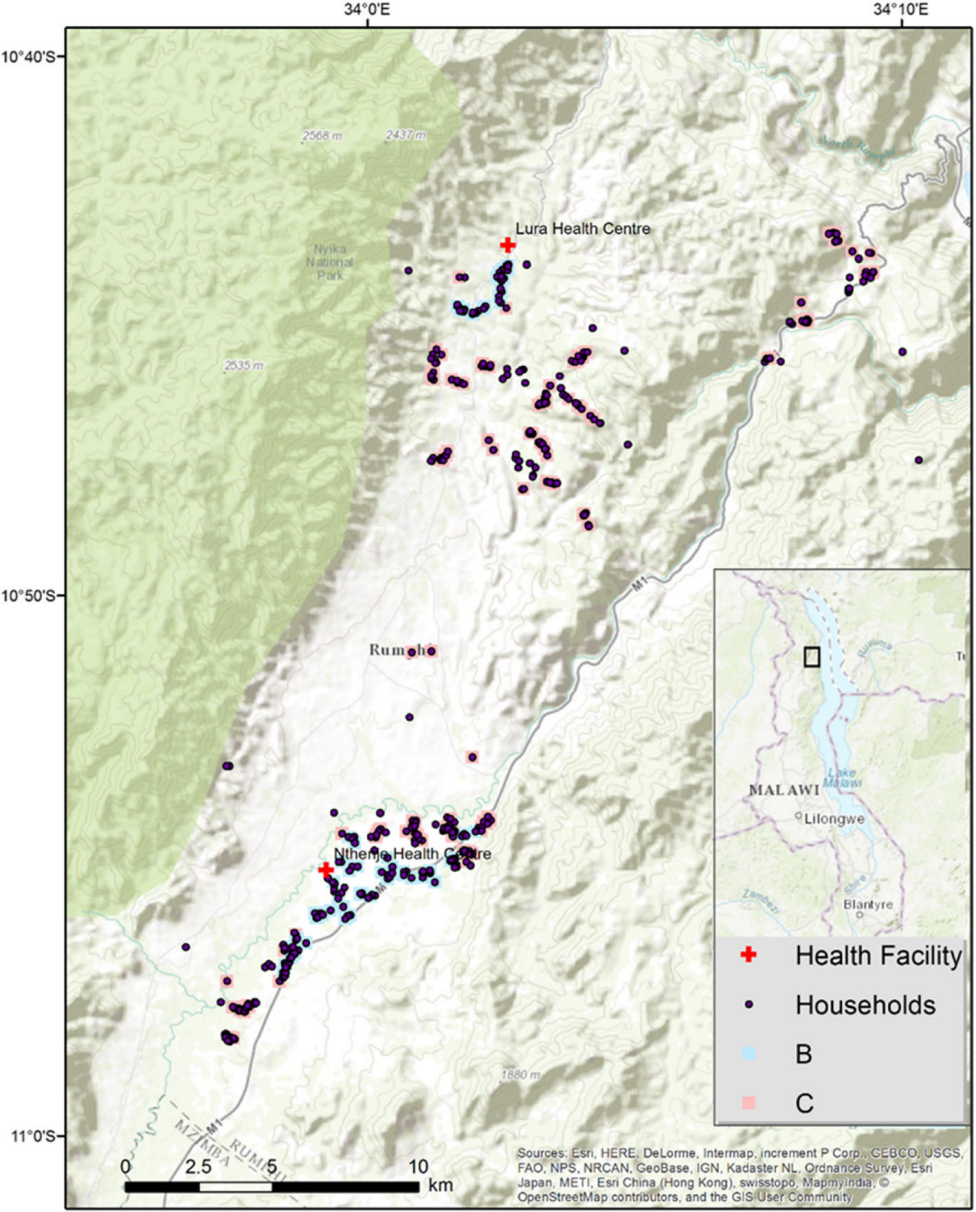
Classification of households based on perceived and measured access, Rumphi, Malawi. *Note:* B and C refer to classification in Table 1 (divergence between perceived and measured access)

Qualitative research methods will be utilized to gain more in-depth data on the causes of divergence (outcomes B and C in Table 1), and to better understand how a combination of individual and contextual factors can ameliorate or aggravate access to health care. Focus Group Discussions (FGDs) and Key Informant Interviews (KIIs) will be used to collect data among health workers, other centrally placed professionals and decision makers and health service users with one or more of the vulnerability characteristics described above. The qualitative component of the study will draw on experiences from participatory GIS [47], where the starting point for the discussion is one or more maps created for the purpose. In our case, one such starting point will be a map visualising different outcomes of physical access and perceived access (Fig. 2). However, we expect that map-literacy will be low among many of our informants, so we will use a combination of map-drawing (Fig. 1) and interpretation of printed maps. When using qualitative methods in health geography, the focus is less on measurement, and more on “the interpretation and understanding of ill-health, disease and disability in the context of place” ( [48], p. 100). This data is essential in order to explore and support the quantitative and cartographic data [41].

### An illustration of the approach

Preliminary analyses from Malawi illustrated the potential of this empirical approach. Figure 2 is based on preliminary analyses of a combination of *Equitable* survey data, GPS coordinates of households and health facilities. The map shows households selected in the Rumphi district in northern Malawi. It identifies clusters of households that are classified within the categories B and C from Table 1 above, i.e., households where the self-reported access is worse than average, but measured access is better than average (measured access is better than perceived access; B), and vice versa (measured access is worse than perceived access; C). The clusters were identified by regressing age, health status and household distance to health facility on perceived access. The predicted residuals from this regression were then used to categorise the households into categories A-D. Going ahead, we will also want to explain the variation in other relevant dependent variables including (but are not limited to) local prevalence of disability and barriers to access. Statistical techniques include traditional regression analysis, but also more explicitly spatial modelling tools such as spatial regression analysis and spatial clustering techniques based on Getis-Ord Gi* local statistics. The latter technique will enable us to determine where there are *significant* B and C category clusters of households (Fig. 2).

According to the map, there are households, particularly in the southern part of the region, that are located very close to a health centre, and yet report of poor access. Similarly, we find that whether transport represents a barrier or not vary widely among individuals living in the same area and within the same distance from the road and the facilities. Within small geographical areas where households are close to each other and distance to the nearest health facility is the same for everyone, that lack of transport as a barrier ranges from “insurmountable problem” to “no problem” among those who live in this village. This indicates a variation in access that we assume stems from variation in personal and socio-demographic characteristics in this population.

## Discussion

A mix of individual and contextual characteristics could help explain the observed variations in access. For example, income and personal network may influence available modes of transportation. For a person with relatively good health, having to walk to get to the nearest road might not be an obstacle, while for a person with disabilities or chronic illness, it could effectively deter access. Hence, the interaction between individual and contextual characteristics should be taken into account, as individual factors of vulnerability may moderate or mediate the impact of physical barriers on access, and vice versa. The novel combination of survey data, geographical data and qualitative exploratory data in *GeoHealthAccess* will generate a model on access to health services in poor contexts that will feed into efforts to improve access for the most vulnerable people in the most underserved areas of the world. Both the specification of “barrier hotspots” where both perceived and measured access is poor, identification of areas with large divergence between perceived and actual access, and identifying the impact of individual and household vulnerability factors such as disability on access has the potential to guide the targeting of resource input such as out-reach health services. In order to ensuring utilization of the findings of this study, the consortium will present the results to officials in the MoH in Malawi and Namibia.

## Conclusion

Given the large number of people who die or become disabled from largely preventable diseases in Africa, as seen in low life expectancy and high level of child mortality and child disability, there is an urgent need to increase access to basic health services, in particular among the most vulnerable groups. *GeoHealthAccess* will provide knowledge that can be applied to developing efficient health policies that target barriers to access both at the community level and at the individual level, improving access where it is most needed.

## Consortium

*GeoHealthAccess* is a collaborative project between SINTEF Technology and Society, University of Malawi, University of Namibia, and Norwegian University of Science and Technology. Stellenbosch University, South Africa, University of Cambridge and University of East Anglia, UK, Trinity College Dublin and University College Dublin, Ireland, and Peace Research Institute Oslo, Norway, are all involved as advisors to the project. The project is funded by the Research Council of Norway, while EquitAble was funded by EU/FP (European Union/Framework Program) 7.

## Abbreviations

EU: European Union
FGD: Focus group discussion
FP: Framework program
GIS: Geographical information systems
GPS: Global positioning system
KII: Key informant interview
WDR: World Disability Report
WHO: World Health Organization

## Footnotes

1 www.Equitableproject.org.

2
https://www.sintef.no/en/projects/geohealthaccess-the-geography-of-vulnerability-and/
